# The Retinoid and Non-Retinoid Ligands of the Rod Visual G Protein-Coupled Receptor

**DOI:** 10.3390/ijms20246218

**Published:** 2019-12-10

**Authors:** Joseph T. Ortega, Beata Jastrzebska

**Affiliations:** Department of Pharmacology and Cleveland Center for Membrane and Structural Biology, School of Medicine, Case Western Reserve University, Cleveland, OH 44106, USA

**Keywords:** G protein-coupled receptor, flavonoids, opsin, retinoids, rhodopsin, small molecules

## Abstract

G protein-coupled receptors (GPCRs) play a predominant role in the drug discovery effort. These cell surface receptors are activated by a variety of specific ligands that bind to the orthosteric binding pocket located in the extracellular part of the receptor. In addition, the potential binding sites located on the surface of the receptor enable their allosteric modulation with critical consequences for their function and pharmacology. For decades, drug discovery focused on targeting the GPCR orthosteric binding sites. However, finding that GPCRs can be modulated allosterically opened a new venue for developing novel pharmacological modulators with higher specificity. Alternatively, focus on discovering of non-retinoid small molecules beneficial in retinopathies associated with mutations in rhodopsin is currently a fast-growing pharmacological field. In this review, we summarize the accumulated knowledge on retinoid ligands and non-retinoid modulators of the light-sensing GPCR, rhodopsin and their potential in combating the specific vision-related pathologies. Also, recent findings reporting the potential of biologically active compounds derived from natural products as potent rod opsin modulators with beneficial effects against degenerative diseases related to this receptor are highlighted here.

## 1. Introduction

### GPCRs as Drug Targets

The G protein-coupled receptors (GPCRs) are highly conserved and ubiquitously expressed in the cellular membrane of eukaryotes. GPCRs provide ways for cells to sense and respond to their external environments through various ligands such as neurotransmitters, peptides, lipids, amino acids, nucleotides, light, and odorants. These receptors are involved in maintaining the organism’s homeostasis by regulating a plethora of physiological processes [1]. GPCRs have been grouped into six classes (namely A to F) mostly based on their sequence homology, but also structural and functional properties [2]. GPCRs comprise of seven alpha-helical domains embedded within the cellular membrane that are connected by three extracellular loops (ECLs) facing the extracellular milieu and three intracellular loops (ICLs) facing the cytoplasm. The N-terminus of GPCRs is located outside and the C-terminus inside the cell. Their ligand-binding pocket, a hydrophobic cavity, able to accommodate a specific ligand is located on the extracellular side of the receptor [3]. The binding of a ligand triggers conformational changes within the receptor and its transition to the active state stimulating the specific G protein mediator, and thus signal transduction [4]. GPCRs also participate in many pathophysiological processes, thus they are attractive targets for the development of pharmaceutical therapeutics. The majority of drugs targeting GPCRs are orthosteric agonists or antagonists that compete with the endogenous ligands. Between 30–50% of drugs approved by the Food and Drug Administration (FDA) target GPCRs [5]. Importantly, only approximately 18% of the identified non-olfactory GPCRs are targeted by these FDA-approved drugs [3]. The most frequently targeted GPCRs in terms of the number of available drugs are serotonin, histamine, adrenergic, and opioid receptors. However, pharmacological modulation of more than 56% of GPCRs remains largely unexplored mostly due to their complex pharmacology and limited experimental tools that constrict the satisfactory progress of this research area. Knowing that these not-targeted GPCRs could be associated with pathologies stresses the necessity of advanced screening and identification of novel GPCR ligands [3,5,6]. Despite these challenges, important advances in GPCR drug developments have been made in recent years that are related to enhanced availability of structural information. The increasing number of GPCR X-ray crystal structures, structures emerging from cryoelectron microscopy (cryoEM), and advances in computational modeling enabled identification of new allosteric binding sites in GPCRs, and thus opened a possibility for discovery of new GPCR allosteric modulators [7,8,9]. Understanding the complex pharmacology of GPCRs is of great interest to biology in general and pharmaceutical industry in particular. 

## 2. Rhodopsin as a Drug Target 

### 2.1. Rhodopsin Structure and Signaling

Rhodopsin is a class A GPCR abundantly expressed within the specialized rod outer segment (OS) disc membranes of the rod photoreceptor cells. Synthesis of this receptor begins in the inner segments of the photoreceptors where it maturates within the endoplasmic reticulum (ER) and Golgi apparatus before it is transported through the cilium to the OS. The C-terminal tail of rhodopsin contains the motif that binds to cargo proteins involved in this transport. Moreover, rhodopsin’s C-terminus contains also two palmitoylation sites at cysteine 322 and 323 and several phosphorylation sites important for the signaling and regulation of this receptor [10,11]. The N-terminus contains two N-glycosylation sites at asparagine 2 and 15 important for protein maturation and its export from the secretory pathway to the membrane [11]. 

**Figure 1:**
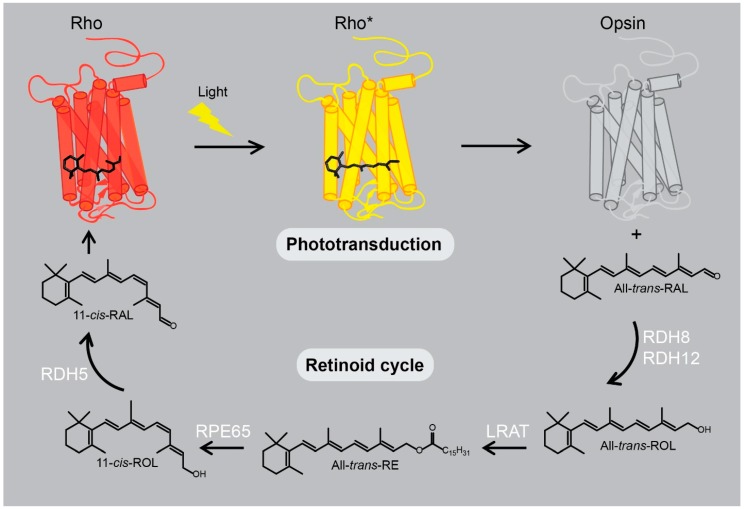
Schematic representation of rhodopsin photoactivation and regeneration. Light illumination triggers isomerization of rhodopsin’s (Rho) chromophore 11-*cis*-retinal (11-*cis*-RAL) to all-*trans*-retinal (All-*trans*-RAL) and transition of the receptor to its photoactivated state (Rho*). Eventually, all-*trans*-retinal dissociates from the retinal-binding pocket resulting in formation of ligand-free opsin and free all-*trans*-retinal, which is reduced to all-*trans*-retinol (All-*trans*-ROL) by retinal dehydrogenases (RDH8 and RDH12). All-*trans*-retinol is then esterified by lecithin retinol acyltransferase (LRAT) to all-*trans*-retinyl esters (All-*trans*-RE) that can be stored in retinosomes or converted to 11-*cis*-retinol (11-*cis*-ROL) by retinyl pigment epithelium-specific protein 65 (RPE65) isomerase. Then, 11-*cis*-retinol is further reduced by RDH5 to 11-*cis*-retinal that re-associates with opsin forming the visual pigment, rhodopsin.

Rhodopsin transmits the signal of light across the biological membrane resulting in the cognitive responses in the brain. This visual receptor is composed of an apo-protein, opsin, and its natural ligand, 11-*cis*-retinal (Table 1), covalently attached to Lys296 via a Schiff base bond. Absorption of a photon triggers chromophore isomerization from 11-*cis* to its all-*trans* configuration, consequently leading to conformational changes, allowing the receptor to adopt its activated state, called metarhodopsin II (Meta II). Meta II enables binding of its cognate G protein transducin (Gt), and thus signal transduction. Eventually, rhodopsin signaling is desensitized through two principal negative regulators: the specific kinase GRK1 that phosphorylates rhodopsin and arrestin that binds to phosphorylated rhodopsin. Subsequently, all-*trans*-retinal dissociates from the chromophore-binding pocket resulting in the formation of unliganded opsin and free all-*trans*-retinal. In the cytoplasm of photoreceptors, all-*trans*-retinal is reduced to all-*trans*-retinol that is then transported to the retinal pigmented epithelium (RPE) cells through the inter-photoreceptor matrix coupled to an inter-photoreceptor retinal-binding protein (*IRBP*). In the RPE all-*trans*-retinol is esterified by the lecithin retinol acyltransferase (*LRAT*) enzyme followed by its de-esterification and isomerization by retinal pigment epithelium-specific protein 65 (*RPE65*) isomerase to 11-*cis*-retinol, which then is oxidized to 11-*cis*-retinal. 11-*cis*-Retinal with the help of *IRBP* is transported back to the photoreceptors, where it spontaneously re-associates with opsin forming functional rhodopsin. This process is called the visual (retinoid) cycle. Such continuous conversion of all-*trans*-retinal to 11-*cis* conformer is critical to maintaining vision (Figure 1).

### 2.2. Rhodopsin’s Orthosteric Binding Pocket - the Retinal Chromophore-Binding Pocket

**Figure 2:**
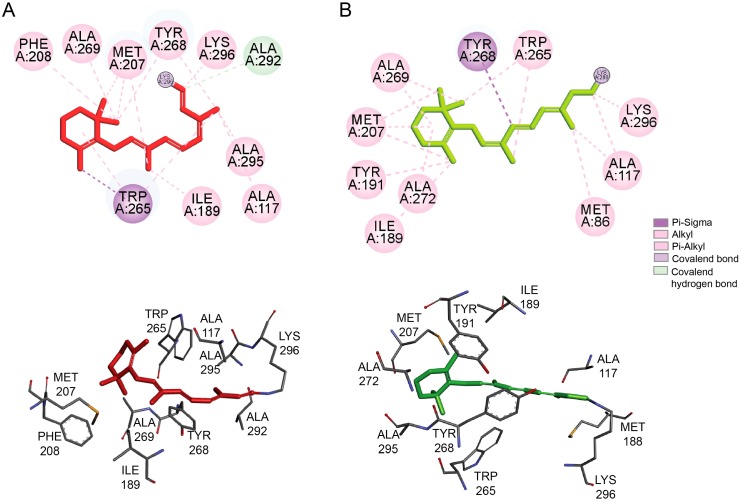
Retinal-binding site. Shown is the configuration of the residues present within the retinal-binding pocket interacting with 11-*cis*-retinal within dark-adapted rhodopsin (**A**) and with all-*trans*-retinal within photoactivated rhodopsin (**B**). The upper panels show 2D diagrams for the main interaction networks between the retinal and the residues within the binding pocket. Specific types of interactions are indicated in the legend. The lower panels show the 3D configuration of the retinal and the residues within the retinal-binding pocket. The light-stimulated isomerization of 11-*cis-*retinal to all-*trans*-retinal triggers the conformational changes within the protein leading to the rearrangement of the residues within the binding site. The coordinates derived from the X-ray structure of dark state rhodopsin (PDB ID: 1GZM) and photoactivated rhodopsin (PDB ID: 4A4M) were obtained from the Protein Data Bank. The co-crystallization products were removed and the resulted PDB files were opened with the VINA/VegaZZ 3.1.0.21 software [13] and the hydrogen atoms and partial charges were assigned to all atoms. Then, the obtained protein structures were optimized with the NAMD 2.12 software [14], applying CHARMM22 forced field [15]. The final structures were visualized with the Biovia Discovery Studio Visualizer 17.2.0 software and 2D diagrams and 3D representations were obtained by selecting the main atoms that interact with the retinal ligand.

The chromophore-binding pocket of rhodopsin is located on the extracellular side of the receptor buried within the transmembrane (TM) domain between the TM helices and enclosed by the ECL2 forming a ‘plug’ protecting the hydrophobic 11-*cis*-retinal from the extracellular environment [10,12]. The chromophore is surrounded mainly by the hydrophobic residues that stabilize the beta-ionone group and the retinal backbone in the *cis*-conformation. The retinal beta-ionone interacts with the residues located within TM5, Met207 and Phe208; and TM6, Trp265 and Tyr268. The retinoid backbone interacts mainly with Ala117 located in TM3, Ile189, located in ECL2 and Tyr268, Ala292 and Ala295, located within TM6 and TM7, respectively (Figure 2A). These interactions rearrange after photoactivation and movement of TMs triggered by the isomerization of 11-*cis*-retinal to its all-*trans* conformation (Figure 2B). 

**Figure 3:**
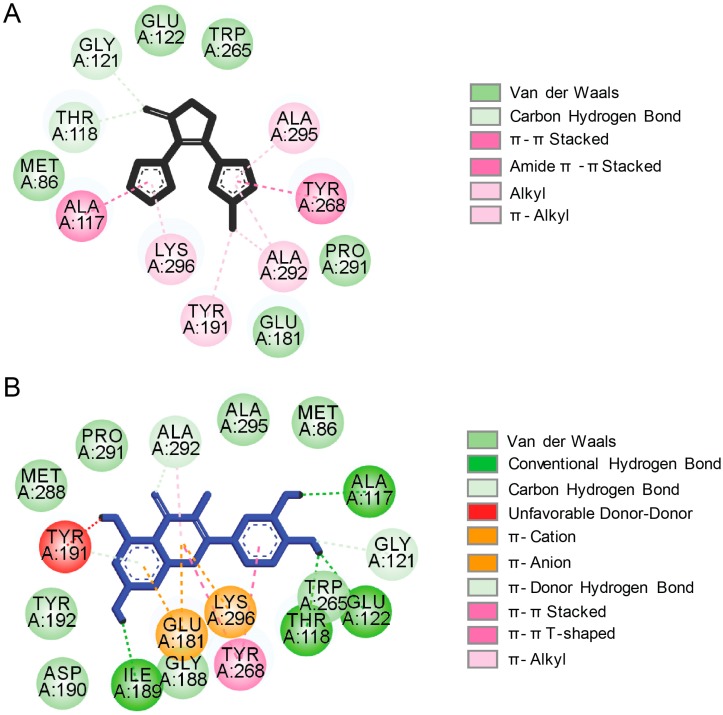
Bioinformatic analysis of the interaction between bovine rod opsin and non-retinoid small molecules. Shown are the molecular docking 2D diagrams of the interactions between rod opsin and YC-001 (**A**) and quercetin (**B**) within the orthosteric binding pocket. YC-001 and quercetin could accommodate within the retinal-binding pocket. However, the interaction pattern and the types of interactions that occur in the orthosteric site are different for these compounds. The molecular docking of YC-001 or quercetin to bovine rod opsin (PDB ID: 3CAP) was performed using VINA/VegaZZ 3.1.0.21 software as described in Ortega et al. [20] with 30 iterations for each compound. The resulting complexes were visualized with the Biovia Discovery Studio Visualizer 17.2.0 software and 2D diagrams were obtained by selecting the main atoms that interact with the ligand.

The orthosteric (retinal) binding site in the apo-protein opsin features a theoretical solvent accessible surface of 495 Å. Despite the natural ligand 11-*cis*-retinal, this hydrophobic cavity also fits other retinoid analogs [16,17,18]. Moreover, the non-retinoid ligands consisting of aromatic rings can accommodate the retinal-binding pocket and interact with the surrounding residues via van-der-Waals and π–π stacking interactions [19,20]. The interaction profiles of rod opsin with YC-001 (Table 1), a recently identified small molecule chaperone that improves membrane targeting of retinitis pigmentosa (RP)-linked P23H opsin mutant, and flavonoids (Table 1) emerged from our in silico analyses are shown in Figure 3A,B, respectively. Interestingly, these structurally different compounds accommodate within the chromophore-binding pocket in close proximity to Trp265, similarly to the retinal. However, obtaining the crystal structures of opsin with these non-retinoid compounds bound would be necessary to validate the computational predictions and this is of high interest. 

### 2.3. Potential Rhodopsin Allosteric Binding Pockets 

**Figure 4:**
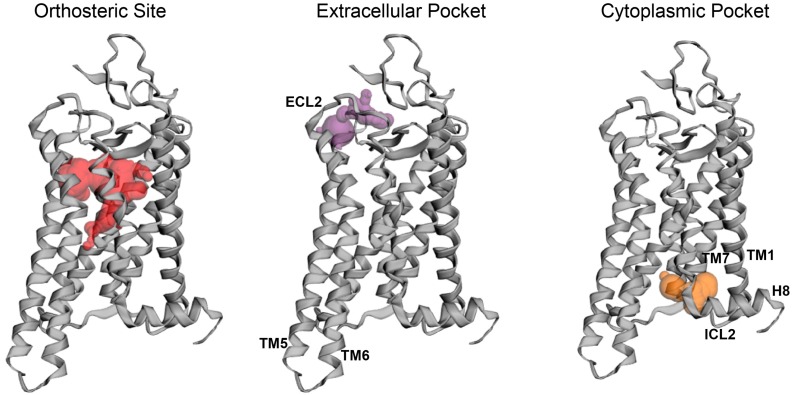
Topological analysis of the potential binding sites for non-retinoid small molecules within the bovine rod opsin structure (PDB ID: 3CAP). The surface analysis was performed with the CASTp 3.0 software [31]. Three main pockets related to protein–drug interaction were identified: (1) the orthosteric site (shown in red), (2) the extracellular pocket between TM5, TM6, and ECL2 (shown in purple), and (3) the cytoplasmic pocket between TM1, TM7, ICL2, and H8 (shown in orange). The crystal structure of ligand-free bovine opsin (PDB ID: 3CAP) was processed as described in Figure 2. Then, the surface topology analysis was performed by using the CASTp 3.0 software available at http://sts.bioe.uic.edu/castp/server3.

The allosteric modulation of GPCRs gained high interest within the last years [3,21,22]. GPCRs are targeted by over one-third of currently FDA-approved drugs. However, due to the high homology between GPCR subtypes, available drugs feature low selectivity and often cause high off-target activation leading to unwanted side effects [1,3,23]. Thus, the design of allosteric modulators that do not interact with highly conserved regions of the receptor, avoiding the cross-activation between homologous receptors, became a new approach in the development efforts of drugs targeting GPCRs. Such allosteric modulators can either enhance or inhibit the response of the natural ligand only in its presence, eliminating the potential receptor over-activation. Topological analysis of multiple GPCR structures using computational algorithms revealed a high diversity of the allosteric binding sites as the ligands could locate within the ECLs, between TM helices and within the ICLs (see Figure 4 and [23]). Similarly to other GPCRs [24,25], allosteric modulation of rhodopsin also was suggested [26,27,28,29]. Structural analysis of dark state rhodopsin, its photoactivated Meta II state and opsin suggested that the allosteric sites emerge only after light-induced rearrangement of TM helices within the Meta II and opsin structures and involve either rhodopsin’s ICLs or ECLs. In the cytoplasmic region of the receptor, the allosteric binding site localizes between TM1, TM2, and TM7. This ligand binding pocket exhibits a solvent-accessible area of 65 Å and involves the following residues: Thr58, 62 of TM1, Leu68, and Asn73 in ECL1, and Leu76, 77 of TM2, as well as Tyr306, Ile307, Asn310, Gln312, and Phe313 of TM7. Meanwhile, in the extracellular region of the receptor, the binding site involves the residues within the ECL2 and tops of TM5 and TM6. This pocket exhibits a solvent-accessible area of 166 Å and is formed by the following residues: Asn2, 200; Gly3, 280; Tyr10, 191, 192; Pro12, 180, 194; Glu181, 201; Gln184, 279; Thr193, Val 204, Ala272, Ile275, Phe276, 283; Asp282; and Met288 (Figure 4). Interestingly, natural products-derived polyphenolic compounds such as anthocyanin and flavonoids were identified as rhodopsin’s allosteric modulators that change the rates of rhodopsin regeneration or Gt binding and signaling activation. As suggested, anthocyanins accommodate the cytoplasmic allosteric binding site in rod opsin [28,29], while flavonoids bind rather to the extracellular region of this receptor [17,24]. As noted, flavonoids also improve stability, folding and membrane targeting of RP-linked specific rod opsin mutants [20]. The beneficial effects of polyphenolic compounds enhancing night vision have also been reported [30]. Thus, allosteric modulation of rhodopsin could have a positive impact on the development of therapies against retinal degenerative diseases. In addition, a combination of the natural products with the retinal supplementations (see below) could potentially achieve an even greater therapeutic outcome. 

## 3. Eye-Related Pathologies Associated with Defective Synthesis of Retinal Chromophore

### 3.1. Leber Congenital Amaurosis 

Dysfunction of the visual cycle key enzymes disables the regeneration of 11-*cis*-retinal from free all-*trans*-retinal accumulated in the retina upon photoactivation, and thus the formation of functional rhodopsin. For example, mutations in the *LRAT* enzyme and *RPE65* isomerase lead to Leber Congenital Amaurosis (LCA), a severe early-onset blinding disease, which accounts for ~5% of all inherited retinal dystrophies. 

#### Retinoids as Pharmacological Supplements for Treatment of LCA

Dietary supplementation with 9-*cis*-retinal (Table 1) has been proposed to bypass the absence of the *LRAT* and *RPE65* unique enzymes and overcome the biochemical deficiency as this isochromophore is more stable and easier to synthesize than the natural ligand, 11-*cis*-retinal. 9-*cis*-retinal binds to unliganded opsin forming photosensitive, functional receptor enabling visual perception. Although 9-*cis*-retinal is absorbed well in the gastrointestinal tract, it is also rapidly cleared within 1–2 h, indicating that frequent drug administration would be necessary to maintain its effect. During further drug optimization, 9-*cis*-retinyl acetate was identified as a useful nutritional supplement restoring vision in animal models of *LCA*. Oral administration of this retinoid to *RPE65^-/-^* or *LRAT^-/-^* mice led to the formation of isorhodopsin, detected by UV-vis spectroscopy in the protein sample purified from the rod OS membranes isolated from mouse eyes, and restored visual function, as determined by electroretinography (ERG). Interestingly, 9-*cis*-retinyl acetate upon ingestion is converted to the prodrug, which is stored in the liver and transported to the eye, where it also can be stored in lipid droplets called retinosomes. This artificial precursor of the retinal chromophore can be converted to 9-*cis*-retinal and used to combine with opsin to form isorhodopsin. To overcome the fast clearance of this retinoid, chitosan-9-*cis*-retinal conjugate was developed [32]. From this conjugate, 9-*cis*-retinal is slowly released in the gastrointestinal tract, assuring lower and more constant levels of this retinoid in the plasma, avoiding potentially toxic levels of 9-*cis*-retinal in the circulation. In addition, the slow release of 9-*cis*-retinal over a longer period would provide a longer-term effect [32]. Vision could be reestablished in both *LRAT^-/-^* mice and dogs carrying *RPE65^-/-^* mutation treated with chitosan 9-*cis*-retinal conjugate [32]. Alternatively, 11-*cis*-6-membered-ring-retinal, an analog of 11-*cis*-retinal that upon binding to opsin is locked within the chromophore-binding pocket even after photoactivation, was shown to rescue visual acuity in *LRAT^-/-^* mice [16,32]. Despite these positive effects, long term studies are still necessary to determine the toxicity of these compounds in humans.

### 3.2. Age-related Macular Degeneration 

Age-related macular degeneration (AMD) is a leading cause of irreversible blindness in industrialized countries. Difficulty with night vision and impaired rod cell-mediated dark adaptation typifies this condition [33,34,35,36,37,38]. Abnormal dark adaptation kinetics is associated with the accumulation of lipofuscin and drusen at Bruch’s membrane, which might create a diffusional barrier to 11-*cis*-retinal and difficulty to reach the photoreceptors, slowing the rates of rhodopsin regeneration [34,36,39]. Consequently, with age the ratio of free opsin to rhodopsin in the rod photoreceptors increases. Excessive accumulation of constitutively active opsin that spontaneously activates signal transduction in photoreceptors accelerates retinal degeneration similarly as observed in LCA. In addition, increased demand for ATP in such condition associates with enhanced consumption of oxygen, which results in increased formation of toxic free radicals and eventually drusen. Thus, inhibition of opsin endogenous activities could be one potential therapeutic approach to prevent or slow down degenerative processes in the retina during aging.

#### Retinoids as Pharmacological Supplements for Treatment of AMD

Treatment with 9-*cis*-retinyl acetate and/or 11-*cis*-6-membered-ring locked retinal could be a potentially useful approach in human conditions associated with delayed regeneration of rhodopsin. Ligand-free opsin accumulated in such a condition that likely initiates AMD pathology could be attenuated by administrating *cis*-retinoid prodrug. Indeed, long-term monthly administration of 9-*cis*-retinyl acetate to C57BL/6 mice for up to 14 months of age significantly improved the function of declining photoreceptors as compared to non-treated mice [40]. A lower opsin/rhodopsin ratio and an increase in the dark adaptation rate were also noted. Thus, treatment with 9-*cis*-retinyl acetate could be a potential therapeutic strategy to prevent age-related retinal deterioration and dysfunction. 

Furthermore, as reported in another study, intraperitoneal (i.p.) injection of 11-*cis*-6-membered-ring-retinal to *Abca4^-/-^Rdh8^-/-^* mice that resemble many hallmarks of AMD and its juvenile form, Stargardt disease, prior to light illumination prevented the retinal degeneration in these mice. These mice, lacking two genes important for retinoid clearance, the *ABCA4* transporter and *RDH8* dehydrogenase, exposed to bright 10,000 lux light develop severe retinal degeneration within seven days. Pretreatment with 11-*cis*-6-membered-ring-retinal before illumination mitigated retinal damage, likely due to the immediate quenching of excessive amounts of ligand-free opsin generated upon exposure to bright light [16].

Altogether, the efficient binding of the retinal chromophore to opsin is critical to maintaining a functional receptor enabling visual perception. Thus, pharmacological supplementation with the retinal chromophore is one potential avenue to treat pathologies with impaired or delayed formation of rhodopsin. However, a precise dosage regimen needs to be carefully established first as excessive concentrations of retinoid therapy could imbalance retinoid homeostasis and cause toxicity. 

## 4. Eye-Related Pathologies Associated with Mutations in *RHO* Gene

Mutations in rhodopsin are the most prevalent cause of blinding diseases such as retinitis pigmentosa (RP), due to initial rod photoreceptor degeneration followed by the decline of cone photoreceptors. Early symptoms of RP include night blindness (nyctalopia) and loss of the peripheral vision, which relatively quickly extends into the central visual field resulting in so called tunnel vision. Eventually, also color vision is compromised due to the deterioration of cone photoreceptors. More than 150 mutations identified in the *RHO* gene are associated with autosomal dominant RP (adRP), stationary night blindness (CSNB) or less common autosomal recessive RP (arRP). These mutations cause a variety of defects such as protein instability and misfolding, mistrafficking and disrupted membrane integration, or altered receptor function, resulting in imbalanced homeostasis leading to rod and cone photoreceptors death. 

### 4.1. Retinoids as Pharmacological Chaperones to Treat Retinitis Pigmentosa

Lack of effective medications for RP stresses the urgency of studying the underlying mechanism of this heterogeneous disease and the need for the development of treatments targeting the specific RP-linked rhodopsin mutants. 

Mutation in the amino acids located in the ECL2, conserved residues in TM helices that are involved in the inter-helical interactions, residues in the chromophore-binding site involved in retinal binding and the G protein-binding site disturb the folding and structural stability of rhodopsin. In fact, binding of the native chromophore 11-*cis*-retinal is critical for rhodopsin stability. Indeed, retinal-free opsin is highly unstable and prone to unspecific aggregation and degradation. The temperature of melting of opsin (Tm = 54.4 °C) is significantly lower than that of retinal-bound rhodopsin (Tm = 71.9 °C), indicating that the interaction network between retinal and the specific residues in the binding pocket is critical for the stability of this visual receptor [20,41]. adRP mutations can perturb the chromophore-binding site leading to various degrees of impairments in the binding of retinal, which then can result in improper folding and inefficient membrane integration of rhodopsin. Partially misfolded mutants that bind some 11-*cis*-retinal can be rescued by retinoid-based chaperones that promote correct folding and membrane trafficking. In contrast, mutants unable to bind retinal are arrested in the endoplasmic reticulum (ER) when expressed in vitro in mammalian cells. The advantageous effect of the 11-*cis*-retinal chromophore, 9-*cis*-retinal isochromophore as well as non-isomerisable 11-*cis*-7-ring-membered-retinal has been reported for several partially misfolded adRP mutants, including P23H, the most prevalent RP-causing mutation found in patients in the United States [42,43,44,45]. These retinoids added to the cultured cells expressing opsin mutants early during biogenesis result in the improved folding and their significantly higher levels in the plasma membrane [19]. Consequently, binding of retinoid rescues cells from the toxic effects of misfolded protein, its accumulation, and aggregation in the ER. Thus, these in vitro studies suggested the pharmacological potential of *cis*-retinoids. However as discovered recently, 9-*cis*-retinal added to the cells co-expressing adRP mutants together with WT opsin resulted in the aggregation of the mutant receptor, indicating that treatment with this pharmacological chaperone could be beneficial for the homozygous patient but rather ineffective or detrimental for heterozygous patients [46]. Therefore, screening for safer, non-retinoid small molecules that would stabilize rod opsin and improve its folding is of urgent importance. In the attempt of such effort, a retinoid-based 5,8-epoxy-13-*cis*-retinoic acid (13-*cis*-5,8-ERA) compound (Table 1) was recently found by using virtual screening against the retinal-binding site [47]. This compound not only fits opsin’s orthosteric binding pocket well, but also possesses high structural similarity to 11-*cis*-retinal. 13-*cis*-5,8-ERA likely binds into the retinal-binding pocket in vitro and competes with 9-*cis*-retinal, but it cannot form a Schiff base bond with apo-opsin. Moreover, its binding efficacy is multiple folds lower than that of 9-*cis*-retinal. Preincubation of opsin membranes with 13-*cis*-5,8-ERA slows rates of pigment regeneration and formation of isorhodopsin. Interestingly, its effectiveness towards several adRP mutants, including the P23H opsin, for increasing opsin mobility from the ER to the plasma membrane was greater than 9-*cis*-retinal. However, the efficacy of this compound and its therapeutic potential still needs to be confirmed in vivo, in model animals.

### 4.2. Non-retinal Small Molecule Modulators of Retinitis Pigmentosa-Linked Rhodopsin Mutants

Currently, the treatment of patients carrying adRP mutations in the *RHO* gene is limited. Retinoid-derived pharmacological chaperones, despite their positive effects in vitro, can risk imbalanced retinoid homeostasis in the retina and exacerbate photoreceptor death [48]. Thus, identification of non-retinoid small molecules that would improve the stability of partially misfolded opsin mutants is an ongoing effort of multiple research laboratories. A novel small molecule chaperone of rhodopsin, YC-001 with agonist-like binding and non-competitive antagonist activities towards rod opsin, was recently identified using high-throughput screening (HTS) [19]. YC-001 does not form a Schiff base with opsin apo-protein. However, it binds to opsin competitively with 9-*cis*-retinal when preincubated with opsin prior to 9-*cis*-retinal. In addition, when bound to rod opsin, it slows down the kinetics of opsin regeneration with 9-*cis*-retinal. Importantly, YC-001 significantly improves mobilization of adRP opsin mutants, including P23H opsin, from ER to the plasma membrane in NIH3T3 cells stably expressing these mutants, even in cells exposed to light, while treatment with 9-*cis*-retinal has to be performed in the dark. Furthermore, upon i.p. administration to *Abca4^-/-^Rdh8^-/-^* mice susceptible to acute light damage, YC-001 protected retinas against light-induced retinal degeneration, likely due to the stabilization of free opsin generated after light exposure and silencing its constitutive activity detrimental to the photoreceptors’ health. Thus, YC-001 could serve as a novel non-retinoid drug candidate modulating opsin and improving its stability.

Also, other non-retinoid opsin modulators (RS1-4) were recently identified by using a structure-based design approach, selecting ligands based on similarities to the natural ligand and fit to the dark state rhodopsin (PDB ID: 1GZM) [49] or photoactivated rhodopsin (PDB: ID: 4A4M) [50] as criteria for virtual screening of Roche’s compounds collection [51]. These compounds bound to rod opsin and enhanced its stability in vitro. Moreover, the receptor-ligand interactions within the orthosteric binding pocket were determined by co-crystallizing RS1 (Table 1) with human opsin and as found, this molecule penetrated rather deep into the helical bundle towards the cytoplasmic side of the receptor, but it did not reach the proximity of the active Lys296. Moreover, several other RS1 compounds modified through medicinal chemistry and bound to opsin were also crystallized. In addition, compounds RS2-4, but not RS1, improved folding and rescued membrane trafficking of P23H rod opsin in cells expressing this mutant.

Altogether, these limited studies indicate that opsin, similarly to the other class A GPCRs, can accommodate chemically diverse ligands within the orthosteric binding pocket with beneficial effects for the receptor stability, folding and trafficking of pathogenic mutants. 

### 4.3. Natural Products-Derived Compounds as Rhodopsin Modulators and their Potential for Treatment of Retinitis Pigmentosa-Related Retinopathies 

Developing new small molecules advantageous for the specific adRP-linked rhodopsin mutants is at the forefront of current research [19,47,51]. However, caution is necessary as such synthetic drugs, despite their distinct positive effects, may cause toxicity and undesired side effects. Thus, natural products and compounds derived from natural product scaffolds could be a viable alternative to the discovery efforts for new drugs and therapies. Plants contain bioactive compounds that have been proven to have therapeutic effects for various human pathologies [52]. The utilization of plants and plant extracts for medical intervention has a long history and it is still currently practiced. In addition, advances in computational chemistry and increased availability of natural product libraries greatly increased the development of new drugs derived from natural products [53,54]. Interestingly, 15% of the FDA-approved drugs targeting various GPCRs are derived from natural product scaffolds [5]. A diet rich in fruits and vegetables containing polyphenolic compounds has been demonstrated to benefit ocular pathologies, including AMD, glaucoma, and RP [55,56,57,58,59]. These compounds exhibit anti-inflammatory, anti-oxidant, and anti-apoptotic properties (see the next section), but they can also interact directly with rod opsin. 

Recent studies probing the interaction between flavonoids and rod opsin revealed that specific flavonoids such as quercetin and myricetin (Table 1) can bind to ligand-free opsin and act as its structural modulators [20]. Computational analysis of the available crystal structures for the dark state, 11-*cis*-retinal-bound rhodopsin (PDB ID: 1GZM) [49], photoactivated rhodopsin (PDB ID: 4A4M) [50] and ligand-free opsin (PDB ID: 3CAP) [60] detected potential binding modes for flavonoids only in the ligand-free opsin structure. These include a vacant orthosteric binding site and an allosteric binding site on the receptor surface that emerged between TM5, TM6, and ECL2 due to light-stimulated conformational changes. Both the empty retinal-binding pocket and the external binding pocket could accommodate both flavonoids. Binding of flavonoids stabilized opsin. As detected with the thermal shift assay, Tm increased from 55.4 °C characteristic for opsin to 61 °C and ~59 °C upon incubation with quercetin and myricetin, respectively. Subsequent opsin incubation with 9-*cis*-retinal after the binding of flavonoids resulted in a cooperative increase of Tm, to nearly 80 °C. However, flavonoids did not provide any stabilizing effect for the chromophore-bound rhodopsin, confirming that the binding sites for flavonoids appear only in the chromophore-free state of this receptor. Direct interaction of flavonoids with opsin and their stabilizing effect due to increased structural rigidity was also reported for cyaniding-3-glucoside by using ^1^H NMR [29]. Enhanced stability and regeneration with 9-*cis*-retinal were also noted for the adRP-associated unstable mutant G90V [27]. The opsin-stabilizing effect of flavonoids could also be related to the enhanced opsin self-association facilitated by flavonoids. Indeed, rhodopsin assembled into oligomers within the ROS membranes is more stable than dissociated monomers caused upon solubilization with detergent [61,62]. In addition, the binding of flavonoids did not prevent pigment regeneration with 9-*cis*-retinal but rather resulted in increased rate of retinal binding to opsin by about two-fold [20,27]. A similar effect of increased regeneration rates was also observed for polyphenols such as anthocyanin and cyanidin-3-glucoside [26,63]. In another study, cyanidin mono and diglycosides accelerated the regeneration of rhodopsin with native chromophore 11-*cis*-retinal [64]. Interestingly, bound flavonoids improved folding and increased the levels of both adRP-linked P23H opsin and WT rod opsin in the plasma membrane in cultured cells [20]. While an increased plasma membrane concentration of WT opsin was related rather to an increasing protein expression, in the case of P23H opsin, flavonoids enhanced mobility of this mutant through the secretory pathway from the ER to the cell surface. 

Together, these studies suggest that the use of flavonoids or flavonoids in combination with the specific *cis*-retinoids could be a new therapeutic approach against ocular impairments associated with certain adRP-linked mutations in rhodopsin. In addition, the ability to accelerate regeneration of rhodopsin by flavonoids and other polyphenolic compounds might be beneficial for enhancement of rod cell light sensitivity in visual pathologies such as AMD, associated with the compromised pigment regeneration and accumulation of unliganded opsin. 

## 5. Protective Effects of Natural Products in In Vitro and In Vivo Models of Visual Diseases

The natural products-derived polyphenolic compounds exhibit beneficial effects in many pathological conditions, including cancer, cardiovascular diseases, neurodegeneration, and urinary tract problems. They also are involved in the modulation of diverse processes of vision physiology and their positive action for age-related vision impairments have been clinically demonstrated [65]. As described in the previous paragraph, polyphenols such as flavonoids can directly interact with rhodopsin and modulate its stability and function. In addition, they likely function as anti-oxidants. This property is particularly important in the eye, as the retina has the highest metabolic rate, which creates a higher oxygen demand than for any other tissue in the body. Thus, the retina is specifically susceptible to formation of reactive oxygen species (ROS) and oxidative stress implicated in multiple ocular pathologies, including AMD-like degeneration. High content of polyunsaturated fatty acids in photoreceptor membranes prone to oxidation enhance vulnerability to oxidative stress and retinal damage. Changes related to the oxidative stress are specifically prominent within photoreceptors and the RPE cells that recycle retinoids upon rhodopsin photoactivation and are involved in phagocytosis of the tips of photoreceptor outer segments as well as retinoid uptake from blood circulation. These changes accumulate with age and ultimately lead to AMD. Clinical reports indicate that antioxidant diet rich in carotenoids and polyphenolics can reduce progression of vision loss up to 28% in patients with moderate macular degeneration [66].

The effects of flavonoids on the oxidative stress-induced cellular mortality have been evaluated broadly in vitro in the primary RPE-derived immortalized ARPE19 cells and photoreceptor-derived 661W cells subjected to hydrogen peroxide (H_2_O_2_). Selected flavonoids such as kaemferol, nobiletin, eupatilin, hesperetin, and taxifolin showed protective effects against the oxidative insult with EC_50_ in the low micromolar range, increasing cell viability and preventing cellular apoptosis [67,68,69,70]. 

The protective effects of natural products have also been evaluated in vivo in mice models of retinal degeneration induced by exposure to bright light equivalent to the light dose absorbed by the human eye on a sunny day or by illumination with blue light. In such light stress conditions, excess of all-*trans*-retinal and its toxic by-products trigger increased generation of oxygen superoxides and induction of apoptosis, eventually leading to photoreceptors death. Interestingly, these degenerative processes can be quite effectively ameliorated or slowed down by the treatment with natural products [71,72]. For example, xanthohumol, resveratrol, and apigenin-7-diglucuronide prevented retinal damage induced by bright light in BALB/c mice [73,74,75], while quercetin-3-O-α-l-arabinopyranoside suppressed detrimental effects of blue light in these mice, mainly by downregulating of genes triggering apoptosis and inflammation in both stresses [76]. Dietary supplementation with saffron significantly diminished symptoms of continuous exposure to bright light in rats [77]. Moreover, randomized clinical trials suggested that saffron may induce short-term improvement of retinal function in early AMD [78]. Although, the exact mechanism of protection provided by these different natural compounds is not fully understood and requires more thorough studies, most of them, in addition to decreasing the expression of inflammation and apoptosis markers, can scavenge the oxygen free radicals, and thus prevent oxidation of lipids, proteins, and DNA damage, slowing down the neurodegenerative retinal pathologies.

Natural products exhibit also protective effects against inherited neurodegenerative retinal disorders caused by progressive loss of peripheral vision such as RP. For example, curcumin was reported to improve retinal morphology and function in P23H-1 rats by reducing protein aggregation and increased localization of rhodopsin to the outer segments [79]. Moreover, administration of safranal, the main component of saffron, twice a week to P23H-3 rats for four months slowed photoreceptor degeneration and preserved photoreceptor function [80]. Treatment with safranal also prevented secondary degenerative changes in the inner retina of these rats. Indeed, safranal exhibits strong antioxidant activity and is capable of quenching free radicals [80].

The lack of effective treatment to stop the progression of retinal degenerative diseases, or to restore vision after it was lost, increases the urgency for greater efforts toward development of new therapies. Natural products available in diet exhibit a plethora of positive vision preserving effects in a number of ocular pathologies and are well tolerated with high doses. Thus, natural products should be seriously considered as a source of therapeutic agents or a starting point to develop new compounds with greater specificity for their targets. 

## 6. Conclusions and Future Directions

The binding of GPCRs with their specific ligands has been employed as a base to uncover the essential principles of drug-receptor interactions. Continuous development of drugs targeting GPCRs related to various pathologies, including psychiatric disorders, diabetes, thrombosis, Alzheimer’s, and eye-related impairments resulted in a significant increase of drug candidates evaluated in clinical trials in recent years. Additionally, remarkable progress in GPCR pharmacology, high-throughput screening, structural biology, and computational modeling opened new venues for rational drug design and expedited GPCR drug discovery. Moreover, allosteric GPCR modulation promises finding of more selective small molecule therapeutics avoiding outside target activation leading to side effects. In this review, we focused on the visual GPCR, rhodopsin, and its ligands. Over the years, the search for rhodopsin modulators was mainly associated with designing analogs of the natural ligand, 11-*cis*-retinal, capable of interacting within the orthosteric (retinal-binding) site. However, as recently reported, rhodopsin can also interact with non-retinoid small molecules. These findings exposed new opportunities to design and evaluate new allosteric modulators of rhodopsin. Lack of effective medical interventions for blinding conditions related to defects in rhodopsin emphasizes an unmet need to establish new remedies. Natural compounds with therapeutic potential for retinopathies could serve as starting scaffolds for designing new drug molecules-related campaigns. 

## Figures and Tables

**Table 1:** Ligands of Rod Opsin.

Name	Structure	Binding Pocket	References
11-*cis*-retinal	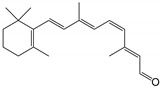	Orthosteric Site	10
all-*trans*-retinal	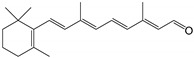	Orthosteric Site	10
9-*cis*-retinal	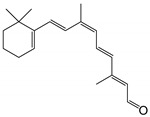	Orthosteric Site	14
11-*cis*-6-membered-ring retinal	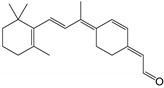	Orthosteric Site	13
5,8-epoxy-13-*cis*-retinoic acid	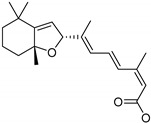	Orthosteric Site	43
YC-001	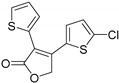	Orthosteric Site	16
RS1	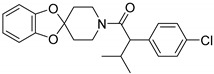	Orthosteric Site	47
Quercetin	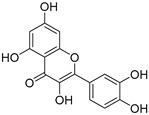	Orthosteric Site Allosteric Site	17, 24
Myricetin	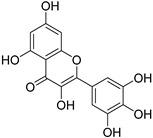	Orthosteric Site Allosteric Site	17
Cyanidin	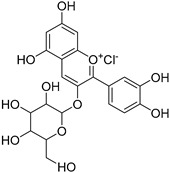	Allosteric Site	26, 27

## Abbreviations

ABCA4	ATP-binding cassette subfamily A, member 4
adRP	Autosomal dominant retinitis pigmentosa
AMD	Age-related macular degeneration
arRP	Autosomal recessive retinitis pigmentosa
ATP	Adenosine triphosphate
CryoEM	Cryoelectron microscopy
CSNB	Congenital stationary night blindness
ECL	Extracellular loop
ER	Endoplasmic reticulum
ERG	Electroretinography
FDA	Food and Drugs Administration
GPCR	G protein–coupled receptor
GRK1	Rhodopsin kinase
Gt	G protein transducin
ICL	Intracellular loop
i.p	Intraperitoneal
IRBP	Interphotoreceptor retinoid-binding protein
LCA	Leber Congenital Amaurosis
LRAT	Lecithin retinol acyltransferase
Meta II	Metarhodopsin II
ONL	Outer nuclear layer
OS	Outer segments
RDH8	Retinol dehydrogenase 8
ROS	Reactive oxygen species
RP	Retinitis pigmentosa
RPE	Retinal pigmented epithelium
RPE65	Retinyl pigment epithelium-specific protein 65
TM	Transmembrane
WT	Wild-type
